# Upgrading an intronic *TMEM67* variant of unknown significance to likely pathogenic through RNA studies and community data sharing

**DOI:** 10.1002/pd.6248

**Published:** 2022-10-21

**Authors:** Alina Kurolap, Adi Mory, Sharon Simchoni, Karina Krajden Haratz, Gustavo Malinger, Roee Birnbaum, Hagit Baris Feldman, Yuval Yaron

**Affiliations:** ^1^ The Genetics Institute and Genomics Center Tel Aviv Sourasky Medical Center Tel Aviv Israel; ^2^ Division of Ultrasound in Obstetrics and Gynecology Lis Maternity and Hospital for Women's Health Tel Aviv Sourasky Medical Center Tel Aviv Israel; ^3^ Sackler Faculty of Medicine Tel Aviv University Tel Aviv Israel

## Abstract

**Fetal phenotype:**

A couple of Ashkenazi Jewish descent was referred for an early anatomy scan at 14 + 2 weeks of gestation following a previous pregnancy termination due to posterior encephalocele and enlarged kidneys. The index pregnancy was also positive for several fetal abnormalities, including enlarged kidneys with cystic dysplasia and abnormal cerebellar morphology highly suggestive of Joubert syndrome.

**Genetic diagnostic test performed, result, and interpretation:**

Trio exome sequencing revealed compound heterozygosity for variants in the *TMEM67* gene: a known pathogenic maternally inherited variant found in *trans* with a paternal intronic variant of unknown significance. RNA analysis revealed that the intronic variant creates a cryptic acceptor splice site in intron 12, leading to the insertion of 22 bp and causing a frameshift with a premature stop codon. This analysis enabled the reclassification of the intronic variant to *likely pathogenic*.

**Implications and novelty:**

This information empowered the couple to make informed reproductive choices and opt for preimplantation genetic testing (PGT) for future pregnancies.

## Key points

**What's already known about this topic?**

- Intronic variants are often classified as variants of unclear significance (VUS).

**What does this study add?**

- This report highlights the importance of RNA analysis in cases where an intronic VUS is found in *trans* with a pathogenic or likely pathogenic variant in an autosomal recessive gene compatible with the fetal phenotype.

## 1 FETAL PHENOTYPE

**FIGURE 1:**
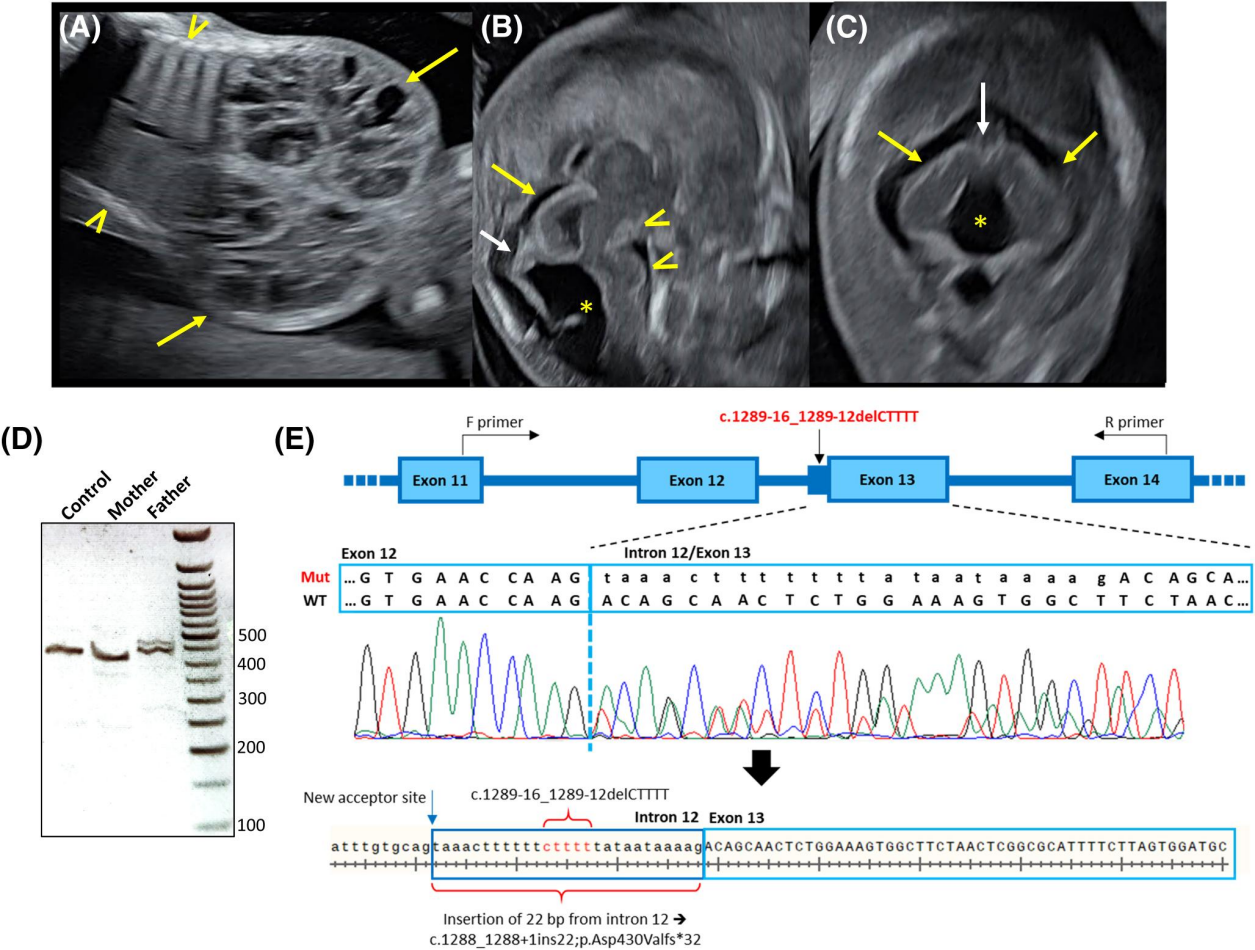
(A–C) Sonographic evaluation at 14 + 2 weeks of gestation: (A) Enlarged kidneys with diffuse cystic dysplasia (arrows). The kidneys fill the whole abdominal cavity when compared to the thorax (arrowheads) and displace the diaphragm upwards. (B) Brainstem has abnormal morphology, kinked and abnormal mesencephalic tegmentum with a deep interpeduncular fossa (arrowheads). (C) Dilated fourth ventricle communicating with the cisterna magna (asterisk) without signs of a primordial intervening vermis (white arrow). The cerebellar hemispheres are displaced and downslanted (yellow arrows). (D and E) RNA analysis and sequencing: (D) Gel electrophoresis of the PCR products amplifying exons 11–14 of *TMEM67* cDNA shows that the paternal intronic variant (c.1289‐16_1289‐12delCTTTT) produces two PCR products—reflecting the wild type allele and a small insertion, while the maternal and the unrelated control cDNA produce only a wild type size allele. (E) The c.1289‐16_1289‐12delCTTTT variant leads to the activation of an upstream cryptic acceptor site in intron 12, subsequently adding 22 bp from intron 12 to the 5′ of exon 13. This intronic addition causes premature termination (c.1288_1288+1ins22; p.(Asp430Valfs*32))

A detailed transvaginal ultrasound examination at 14 + 2 weeks of gestation revealed normal fetal biometrics except for abdominal circumference compatible with 18 weeks. Both kidneys were enlarged filling the entire abdominal cavity and displacing the diaphragm upwards. The renal parenchyma showed diffuse cystic dysplasia with a different pattern from the classic multicystic or polycystic kidney disease (Figure 1A). The urinary bladder remained empty during the evaluation. Neurosonographic assessment identified supratentorial structures compatible with gestational age, but the corpus callosum was not observed as expected. The midbrain and hindbrain examination through the posterior fontanelle showed a dysplastic brainstem and tectum (Figure 1B) and a dilated fourth ventricle communicating with the cisterna magna (Figure 1C). The primordial structure was not identified. These findings were suggestive of Joubert Syndrome, although at this gestational age, the features of the molar tooth sign were not yet present. There was mild nuchal edema with two small cervical cysts. No other anomalies were detected. Clinical data are summarized in Table 1. Of note, a previous pregnancy was terminated at 15 weeks due to a posterior encephalocele and enlarged kidneys.

**TABLE 1:** Clinical data

Case	Parental details			Gestation at diagnosis	Phenotypes (HPO terms)	Obstetric history	Family history	Outcome
1	Maternal	Age	28	14 + 2 weeks gestation	Dilated fourth ventricle HP:0002198; Aplasia/Hypoplasia of the cerebellar vermis HP:0006817; Abnormal brainstem morphology HP:0002363; Fetal nuchal edema HP:0034250; Renal dysplasia HP:0000110	G5P2; two healthy sons, two early miscarriages and one pregnancy termination at 15 weeks due to a posterior encephalocele and enlarged kidneys. No genetic testing; DNA unavailable.	N/A	Pregnancy termination
		Ethnicity	Ashkenazi Jewish					
	Paternal	Age	31					
		Ethnicity	Ashkenazi Jewish					

## 2 DIAGNOSTIC METHOD

Chromosomal microarray revealed a normal male karyotype. Following genetic counseling and informed consent, trio exome sequencing was performed in‐house on a NovaSeq6000 sequencer (Illumina, San Diego, CA, USA). The bioinformatic pipeline was performed using the Franklin data analysis platform (Genoox, Tel Aviv, Israel), as previously described.^1^ The potential effect of the paternal intronic variant on mRNA splicing was studied using parental peripheral blood mononuclear cells (a fetal sample was unavailable). RNA was extracted using the High Pure RNA Isolation Kit (Roche, Basel, Switzerland) and cDNA was synthesized with the qScript cDNA Synthesis kit (Quantbio, Gaithersburg, MA, USA). We used the cDNA to amplify *TMEM67* exons 11–14 for subsequent analyses.

## 3 DIAGNOSTIC RESULTS AND INTERPRETATION

Trio exome sequencing analysis revealed that the fetus was a compound heterozygote for two variants in the *TMEM67* gene (NM_153704.5). The maternally inherited variant (c.1975C>T; p.(Arg659*)) causes premature termination. It has previously been described in Joubert syndrome^2^ and classified as *pathogenic* in ClinVar (VCV001072098.2). The paternally inherited variant is a deletion of 5 bp in intron 12 (c.1289‐16_1289‐12delCTTTT) and was initially classified as a *variant of unknown significance* (VUS). The genetic findings are summarized in Table 2. RNA analysis on the paternal sample revealed two PCR products of different molecular weights: one corresponding with the wild type allele and a larger one reflecting a small insertion (Figure 1D). Upon sequencing, it became evident that the deletion in intron 12 activates an upstream cryptic acceptor spice site, leading to an insertion of 22 bp from intron 12 into the beginning of exon 13, and creating a frameshift with a premature stop codon (p.(Asp430Valfs*32)) (Figure 1E). The variant was, therefore, reclassified as *likely pathogenic* (LP) according to the American College of Medical Genetics and Genomics (ACMG) guidelines.^3^

**TABLE 2:** Genetic findings

Case	Procedure (Gest age)	Performed test	Secondary confirmatory test	Gene (name; REFSEQ)	Known disease (OMIM)	Variant	ACMG classification	Criteria applied	Inheritance and zygosity	Interpretation
1	Post TOP	Exome sequencing	RT‐PCR RNA analysis and Sanger sequencing	*TMEM67* (NM_153704.5)	Joubert syndrome 6 (MIM #610688) and Meckel syndrome 3 (MIM #607361)	Allele 1: c.1975C>T; p.(Arg659*)	Pathogenic	PVS1, PM2, PP5	HET, maternally inherited	Disease causing
						Allele 2: c.1289‐16_1289‐12delCTTTT	Variant of unknown significance, upgraded to likely pathogenic	PS3, PP4, PM3, PM2	HET, paternally inherited	Disease causing

## 4 PREGNANCY OUTCOME

The pregnancy was terminated at 18 weeks. Post‐mortem examination of the kidneys showed diffuse cystic dysplasia.

## 5 DISCUSSION

Our report highlights the importance of pursuing further testing using RNA analysis in situations where a VUS is in *trans* with a P/LP variant in genes compatible with the fetal phenotype, especially in recurrences. In the case described, this resulted in a clear resolution, allowing precise genetic counseling regarding recurrence risk and reproductive options for future pregnancies. Indeed, the couple now opted for preimplantation genetic testing (PGT‐M). Since the submission of this variant classification to the Franklin Community, it has come to our attention that this intronic variant has been detected in *trans* with another pathogenic *TMEM67* variant in two additional fetuses manifesting with a ciliopathy phenotype.

## CONFLICT OF INTEREST

Yuval Yaron is an advisor for Genoox (https://www.genoox.com), developers of the Franklin pipeline used for ES bioinformatics analysis. All other authors report no conflict of interest.

## ETHICS STATEMENT

Exome sequencing was performed as a clinical service under clinical consent forms and RNA studies were performed under a study approved by the Tel Aviv Sourasky Medical Center Institutional Review Board (0276‐11‐TLV). Retrospective collection of data from patient records has been granted a waiver of informed consent, as all clinical data contained in this report have been de‐identified (0039‐15‐TLV).

## Data Availability

The data supporting the findings of this study are available upon request.
